# A standardized core genome multilocus sequence typing and life identification number barcoding framework for global Pasteurella multocida surveillance and outbreak investigation

**DOI:** 10.1099/mgen.0.001733

**Published:** 2026-06-25

**Authors:** Angela V. Lopez Garcia, Keith A. Jolley, Kasia M. Parfitt, Amro Hashish, Yuko Sato, Mohamed El-Gazzar, Javier Nunez-Garcia, Catherine Fearnley, Samuel K. Sheppard, Martin C. J. Maiden, Muna F. Anjum

**Affiliations:** 1 Department of Bacteriology, Animal and Plant Health Agency, New Haw, Addlestone, Surrey, KT15 3NB, UK; 2 Department of Biology, Life and Mind Building, University of Oxford, South Parks Road, Oxford, OX1 3EL, UK; 3 Department of Biology, Ineos Oxford Institute for Antimicrobial Research, Life and Mind Building, University of Oxford, Oxford, OX1 3EL, UK; 4 Department of Veterinary Diagnostic and Production Animal Medicine, Iowa State University, Ames, IA 50011, USA

**Keywords:** core genome multilocus sequence typing (cgMLST), cgMLST-based Life Identification Number (cgLIN) code, global surveillance, *Pasteurella multocida*, population structure, typing scheme

## Abstract

Effective pathogen surveillance requires robust typing systems for outbreak detection and understanding transmission dynamics, evolutionary patterns, host colonization and disease progression. *Pasteurella multocida* is a globally distributed pathogen that infects mammals and birds, causing a variety of diseases. Despite its significance to public and animal health, genotypic characterization of this species remains limited, impeding effective surveillance and control strategies. To address this, we developed a core genome multilocus sequence typing (cgMLST) scheme and a complementary life identification number (LIN) barcoding system for *P. multocida*. The cgMLST scheme was developed and validated using 1,233 core genes identified from 1,593 genomes sourced from more than 8 host species across all continents, spanning collections from 1922 to 2024. Genomes included publicly available assemblies from PubMLST, newly sequenced isolates from the Animal and Plant Health Agency (APHA) and assemblies from Iowa State University. The cgMLST scheme assigned core genome sequence types (cgSTs) to 1,554 of the 1,593 genomes (97.55%), identifying 1,304 unique cgSTs and improving resolution compared to the 2 currently available 7-locus MLST schemes. Based on pairwise allelic differences, 12 thresholds were defined to establish the LIN barcoding system, a hierarchical and stable nomenclature enabling multi-level clustering. Application of the cgMLST-based Life Identification number code to independent outbreak datasets confirmed the scheme’s ability to delineate outbreak clusters and distinguish unrelated isolates. This standardized typing framework provides a robust approach for strain classification, population structure analysis, vaccine target identification and global surveillance of *P. multocida*. Integration into the PubMLST platform ensures open access and facilitates global collaboration in the molecular epidemiology of this pathogen.

**Impact Statement**

*Pasteurella multocida* causes disease across multiple species and regions, leading to significant economic losses and public health concerns. Phylogenetic analyses conducted by laboratories worldwide are often not directly comparable due to methodological differences, and the requirement to exchange raw genomic data hinders rapid international collaboration. For decades, population structure classification has relied on seven housekeeping genes multilocus sequence typing (MLST), a reproducible but limited approach. The seven-gene MLST scheme lacks sufficient resolution to distinguish closely related isolates within outbreak clusters, instead grouping them with distantly related strains. This limitation obscures transmission pathways and delays outbreak response. We present a validated 1,233-locus core genome multilocus sequence typing combined with a 12-level hierarchical life identification number (LIN) barcoding system for high-resolution typing of *P. multocida* populations. This scheme identified 1,304 distinct strain types with 5 five times the resolution of 7-locus MLST, enabling laboratories to accurately differentiate outbreak-related isolates. The LIN code assigned each isolate a unique, directly comparable numerical identifier, enabling real-time international data sharing without exchanging raw genomic sequences. Integration into PubMLST as an open-access resource makes this system immediately available to veterinary and public health laboratories worldwide, facilitating faster, coordinated outbreak detection and response across geographic and jurisdictional boundaries.

## Data Summary

The *Pasteurella multocida* core genome multilocus sequence typing scheme and life identification number barcodes are available in PubMLST (https://pubmlst.org/organisms/pasteurella-multocida). Genome accessions are listed in Table S1. New isolates were deposited in the NCBI under BioProject accession number PRJNA1381339. Supporting data is provided within the article and supplementary files.

## Introduction

*Pasteurella multocida* is a commensal bacterium found in the oral, nasal and respiratory tracts of domestic and wild animals, as well as birds, worldwide [1]. It can also function as a primary or opportunistic respiratory pathogen, which demonstrates capacity for host and geographic transboundary dissemination causing diseases such as fowl cholera in birds, haemorrhagic septicaemia (HS) in cattle, atrophic rhinitis in pigs, snuffles in rabbits and pneumonia in a range of mammals including cattle, pigs, alpacas and sheep [1,4]. In humans, infections typically arise from cat or dog bites, resulting in wound infections that can lead to complications such as respiratory disease, osteomyelitis, meningitis or septicaemia [5,7]. The associated morbidity and mortality in livestock and poultry cause significant economic losses for the farming industry [2,8].

Given this broad impact across species and geographic boundaries, accurate strain classification is essential for epidemiological surveillance and outbreak response. However, achieving this remains challenging, as the validation and implementation of standardized typing schemes capable of reliably translating whole genome sequencing (WGS) data into actionable epidemiological information for surveillance, outbreak investigations and global public health collaboration are still ongoing [9,10].

Over the past two decades, two multilocus sequence typing (MLST) schemes have offered a standardized framework for *P. multocida* strain classification, based on variation at seven housekeeping genes [11,12]. The sequence types (STs) assigned by the MLST can be further grouped into clonal complexes (CCs) based on similarities of allelic profiles [9,13, 14]. Alongside MLST, *P. multocida* typing has also relied on capsular serogroup classification, Heddleston lipopolysaccharide (LPS) serotyping and subspecies designation. Although collectively informative, these approaches each capture only a narrow dimension of genomic diversity. In particular, the slow evolutionary rate of housekeeping genes underlying MLST limits its ability to discriminate between closely related strains involved in local outbreaks or transmission chains [2,15]. Core genome MLST (cgMLST) addresses these limitations by extending analysis to hundreds of conserved core genes, enabling higher-resolution comparisons and improved strain discrimination [9,16,20].

Building upon cgMLST enhanced resolution, this study integrates the life identification number (LIN) system, a taxonomic framework initially proposed by Vinatzer *et al*., that further refines genome classification through hierarchical coding based on cgMLST similarity [21,25]. Each LIN code consists of predefined numbers in sequential ‘bins’, representing ranges of cgMLST profile similarity. Moving from left to right, these bins capture increasing genetic similarity, allowing stable and precise sublineage classification that remains unaffected by the addition of new genomes. Notably, the cgMLST-based Life Identification Number (cgLIN) assigns variants a code that represents genomic relatedness across a spectrum of taxonomic resolution, whereas cgST groups variants at a single resolution level [23,24]. The cgMLST-LIN code approach has been successfully implemented for several clinically important bacterial pathogens [19,23,25], where it was shown to yield a hierarchical nomenclature highly concordant with established CC and ST designations while simultaneously providing greater discriminatory power for outbreak-level investigations.

Here, we present a cgMLST scheme for *P. multocida* based on 1,233 core genes, together with a LIN code system for multi-level classification, developed and validated using isolates from all continents, more than 8 host species and spanning the years 1922–2024. Integration into the PubMLST platform [12] provides an open-access resource for the scientific community and establishes a unified framework for the characterization and surveillance of global *P. multocida* populations.

## Methods

### Culture collection of *P. multocida* isolates

A total of 399 *P*. *multocida* isolates were obtained from the Animal and Plant Health Agency (APHA) archive for this study. These isolates were sourced from bovine (*n*=256), ovine (*n*=46) and porcine (*n*=97) clinical samples, collected in the UK during 2020 and 2023 from scanning surveillance of diseased animals (Table S1, available in the online Supplementary Material). All isolates were recovered from −80 °C bead stocks, cultured on 5% sheep blood agar and incubated aerobically overnight at 37 °C. Species identification was confirmed by MALDI-TOF MS (Biotyper Sirius, Bruker Daltonics, Bremen, Germany).

Additionally, Iowa State University provided 127 *P*. *multocida* genomes from avian hosts and 1 from an unknown host, collected between 2000 and 2021 across North America, South America, Asia and Antarctica (Table S1). DNA extraction and sequencing for these isolates were performed as described in [26].

### DNA extraction and short-read sequencing of *P. multocida*

Genomic DNA was extracted from overnight Luria-Bertani broth cultures of each of the 399 APHA isolates using the MagMax core nucleic acid purification kit and the MagMAX_CORE_DUO heated script in the Kingfisher Flex system (Thermo Fisher Scientific Inc., USA) [27,28]. DNA concentrations were quantified using Qubit 3.0 with the Qubit dsDNA Broad Range Assay Kit (Life Technologies; Thermo Fisher Scientific Inc.). Genomic libraries were constructed using the Nextera XT Library Preparation Kit (Illumina), and short-read WGS was performed on the Illumina NextSeq using 300-cycle kits with paired-end sequencing (151 cycles per read). Species identity of isolates sequenced in this project was confirmed using Kraken2 v1.0 and the Kraken Standard Database [29].

### Genome assembly and annotation

The APHA WGS reads generated in this study were assembled with Unicycler v0.5.1 using default settings [30]. Species identification was performed with fastANI v1.34, classifying isolates with average nucleotide identity >96% as *P. multocida* (Table S2) [31,32]. Assemblies were included in further analyses based on key quality indicators, including absence of multiple alleles per locus in the Rural Industries Research and Development Corporation STs [12] and ribosomal multilocus sequence typing (rMLST) [33]. Assemblies showing multiple alleles per locus in either MLST scheme were excluded as potentially contaminated (Tables S1 and S3). Additional quality metrics included genome length, number of contigs, N50, L50 and GC content as determined by PubMLST (Table S3) [12]. The 1,082 assemblies obtained from PubMLST were cross-referenced with their corresponding NCBI RefSeq submissions, which revealed that some had been flagged for exclusion due to the presence of numerous frameshifted proteins and annotation completeness failures. These flagged assemblies were subsequently removed from further analyses (Table S1) [34].

### Development and validation datasets for core gene identification in *P. multocida*

Genomic datasets were compiled from 1,082 *P*. *multocida* genomes in the PubMLST database (accessed in February 2025), 399 genomes from APHA and 128 genomes from Iowa State University (Table S1). All genomes underwent quality control and were filtered based on inclusion criteria as described above (Tables S1 and S3). Genomes that did not meet these criteria were reviewed and excluded as appropriate.

In total, 1,593 genomes were retained and categorized into 2 datasets. The cgMLST development dataset (*n*=973) comprised all PubMLST genomes available at the time that contained fewer than 200 contigs, ensuring high-quality assemblies suitable for defining the core genome. An additional step confirmed that the 973 development assemblies were sufficient for a stable core genome definition. For this, development dataset assemblies were annotated using Bakta v1.8.2 [35] with the database v5.0 and pangenome analysis was conducted using Roary v3.13.0 [36] with default parameters.

The validation dataset (*n*=620) included PubMLST isolates (*n*=98) that were available at the time the validation analysis was performed, as well as additional isolates from APHA (*n*=397) and Iowa State University (*n*=125), all with fewer than 400 contigs (Table S1). This dataset provided a diverse and independent collection for evaluating the proposed workflows, assessing robustness and validating the cgMLST scheme.

### Core gene identification and curation

Whole-genome sequence data from the development dataset (*n*=973) was used to identify core genes. Coding sequences with in-frame start and stop codons were identified in reference strain *P. multocida* 17BRD-035 (CP082272.1; 2,624,884 bp, 40.23% GC, 344.0× coverage). The BIGSdb ‘sequence tagging’ function in PubMLST [12] was used with the described parameters [14], to detect novel alleles of these reference coding sequences among the development dataset (Fig. S1).

Core genes were identified using the BIGSdb Genome Comparator plugin (v2.8.5), with blastn as the default [12,14]. The initial core gene set was determined as genes present in ≥99% of the genomes, as described previously [20] (Fig. S1).

Manual inspection and curation of core genes were conducted. Loci with truncated or missing alleles, nucleotide variability near the start codon or inconsistent start codons were eliminated. For some loci, start positions were adjusted when the position in the reference sequence was inconsistent across the dataset or when alternative start codons, such as ATA, were identified in close proximity to the originally annotated start codon (Table S4). Additionally, all loci were screened for potential paralogs to ensure unambiguous allele assignment, as multiple gene copies within a genome can result in multiple allele calls for a single locus, potentially compromising cgST assignment. This curation process was essential to optimize scheme performance and ensure reliable automated assignment of novel alleles during continuous scheme updates.

Each gene was assigned a ‘PMUL’ identifier. Finally, cgSTs were assigned to profiles containing up to 25 missing alleles. This threshold is established in the PubMLST software for all the recently developed cgMLST, aiming to balance robustness in cgST assignment while also maximizing the number of isolates that can be assigned a cgST [14,20, 24].

### Validation of the cgMLST scheme

The initial cgMLST scheme was validated on an independent dataset (*n*=620) using the BIGSdb Genome Comparator tool in PubMLST with default parameters [12] (Fig. S1). Genes remaining core after validation were incorporated into the final *P. multocida* cgMLST scheme, which ultimately included 1,233 core genes (Table S5). These core genes were further analysed for allelic variability and intragenic recombination using the pairwise homoplasy index (PHI) in PhiPack with 1,000 permutations [37]. Functional classification was performed with eggNOG-mapper v2.1.11 [14,38], assigning each gene to a Cluster of Orthologous Genes (COG) categories [39] and the KEGG BRITE (Kyoto Encyclopedia of Genes and Genomes) functional hierarchy [14,40]. Core genes classified as ‘Function unknown’ (COG category S) were additionally screened against the Comprehensive Antibiotic Resistance Database (CARD v4.0.1) using Resistance Gene Identifier v6.0.0 [41] and the Virulence Factor Database (VFDB) (December 2025) [42] using Abricate v1.2.0 with default parameters [43] to assess potential antimicrobial resistance (AMR) determinants or virulence factors.

### Exploring population-wide variation in allelic mismatches

We evaluated the population structure of *P. multocida* by analysing allelic mismatches through pairwise comparisons of cgMLST profiles in a subset of 620 genomes (Table S1) selected from the total 1,554 genomes assigned a cgMLST profile to ensure balanced representation of CCs. To assess whether the observed population structure was robust and not dependent on the specific selection of 620 genomes, pairwise comparisons were also performed on two additional subsets of 421 and 901 randomly selected genomes to minimize the subset selection bias (Table S1). Additionally, clustering consistency and robustness to subsampling bias from the subset of 620 genomes were assessed for the full set of 1,554 genomes using the Silhouette index (SI) [44] and the Wallace coefficient (W) [45], as implemented in MSTclust v0.21 with parameter -e 0.90 [46]. To visualize population structure in the 421, 620 and 901 genome subsets, we calculated the percentage of allelic mismatches among genomes and further examined the distribution by grouping them according to shared ST, CC, ribosomal sequence type (rST), the capsular and the LPS types. Six graphs were generated from this, where SI and W were also included. The capsular and LPS types were determined as in [47].

### cgST and LIN code assignment

An LIN code is a multi-position, integer-based identifier where each position (‘bin’) corresponds to a (range of) cgMLST profile similarity value, together representing a partition of the complete range 0–100% [19,24]. Based on the population structure observed in the previous pairwise allelic mismatch analysis, 12 thresholds were defined to delimit 12 bins. These thresholds were incorporated into PubMLST [12], and the LIN code scheme was established. LIN codes were assigned to cgSTs (*n*=1,554). Missing loci are excluded from pairwise allelic distance calculations. To minimize the artefactual reduction of distances between isolates, a threshold of up to 25 missing loci (~2%) was applied for cgST and LIN code assignment, consistent with the standard threshold used on PubMLST for recently developed schemes [23,24]. To evaluate the concordance between cgLIN classification levels (bins) and previously established classification methods, seven-gene MLST, CC, rST, capsular type and LPS serogroups, the adjusted Rand index (ARI) was calculated using the mclust R package [48].

### Core genome alignment and phylogenetic analyses for cgLIN code validation

A subset of 1,554 genomes, each assigned a cgST, was used to construct neighbour-joining trees based on 1,233 core gene sequences, with the aim of testing the congruence between the core genome phylogenetic tree topology and the predefined lineage LIN code threshold. For the construction of the core genome phylogenetic tree, sequences were aligned using MAFFT, with missing alleles treated as gaps and subsequently concatenated, using the iTOL plugin on PubMLST [12]. Congruence was also evaluated between predefined LIN code lineage thresholds and whole-genome phylogeny using a k-mer-based approach with Mashtree using default parameters [49].

### cgLIN code applicability to real outbreak cases

To demonstrate the applicability of the cgLIN code scheme for *P. multocida* global surveillance and outbreak identification, three independent validation analyses were conducted using strains from peer-reviewed outbreak studies (Table S1) [50,52]. The first validation analysis assessed 13 *P*. *multocida* genomes from an outbreak affecting squirrel gliders, woylies and rufous bettongs in an Australian zoo between 2015 and 2017 (Table S1) [50]. Core gene sequences were retrieved and aligned via the PubMLST sequence definition database [12] and visualized with Microreact [53].

The second validation followed a similar core genome SNP phylogeny approach to the original study analysing 102 genomes included in a HS outbreaks study in Germany (Table S1) [51]. ORFs were annotated using Bakta v1.11.0 with database v5.0 [35]. Core and accessory genomes were calculated with Roary v3.13.0 [36], applying a sequence identity threshold of 95% for gene similarity. Core genes were individually aligned using MAFFT v7.526 [54] and were used to construct a maximum-likelihood phylogenetic tree with RAxML-NG v.1.2.2 using the GTR+FO+G4m substitution model [55].

The third validation followed a similar analysis pipeline to the original study for analysing 75 avian-origin *P. multocida* genomes from Australia [52]. Draft genome assemblies were aligned with Parsnp v1.2 [56] with strain X73 (NZ_CM001580.1) as the reference genome. Predicted recombination sites were identified and removed using Gubbins v2.3.4 [57]. A phylogenetic tree based on core genome SNP alignments was constructed using RAxML-NG v1.2.2 with the GTR+FO+G4m substitution model and 1,000 bootstrap replicates [55]. The resulting tree was midpoint-rooted and visualized using FigTree v1.4.4 [58].

## Results

### *P. multocida* genomes from PubMLST, APHA Archives and Iowa State University used in the development and the validation datasets

Distribution analysis (Fig. S2) was conducted to evaluate the spread of key genomic assembly metrics (N50, GC%, number of contigs and genome length) across all assemblies in this study (*n*=1,609). As demonstrated by the violin plots, most assemblies clustered around the median and quartiles, although a notable subset fell outside these ranges (Fig. S2). Assemblies identified as outliers based on these metrics were flagged for further review and, when appropriate, excluded from further analyses (Table S1, Fig. S2). Additional parameters, such as the number of alleles per locus in both the rST and the seven housekeeping gene ST schemes, L50 and quality check failures as indicated by RefSeq, were also considered when flagging assemblies for exclusion (Tables S1 and S3). In total, 16 assemblies were removed: 8 with more than 400 contigs (including 4 with low N50 values, 5,891–17,120, and 2 others with high N50 values, 401,139 and 231,890 but L50 values of 3 and 4, indicating fragmentation), 7 due to a high number of frameshifted proteins or annotation completeness failures according to RefSeq and 1 based on rST quality checks, containing more than 1 allele at 6 loci of the rST scheme, indicating contamination (Tables S1 and S3, Fig. S2). Some assemblies displayed genome length and GC% values outside the typical distribution (Fig. S2); however, these were retained as assemblies with similar metrics have been previously reported in the literature (Table S3).

The final dataset comprised 1,593 genomes: 973 in the development dataset and 620 in the validation dataset for the cgMLST scheme (Table S1). The assemblies have an average genome length of 2,334,828.8 bp (median=2,316,461 bp), with the number of contigs ranging from 1 to 395 (median=35). The average N50 for the remaining 1,593 was 410,518 bp (median=187,058 bp), and the average GC content was 40.31% (Fig. S2).

Further Roary pangenome analysis [36] was performed to confirm that the development set included enough assemblies for robust core gene identification. The *P. multocida* pangenome data indicated this is an open genome, with the total number of genes (red line) increasing as more genomes were added (Fig. S3). The number of conserved core genes (blue line) decreased and stabilized at ~300 genomes, indicating that a stable core genome had been achieved. Thus, it was concluded that the inclusion of 973 genomes was sufficient for a comprehensive core gene identification (Fig. S3).

**Fig. 1.**
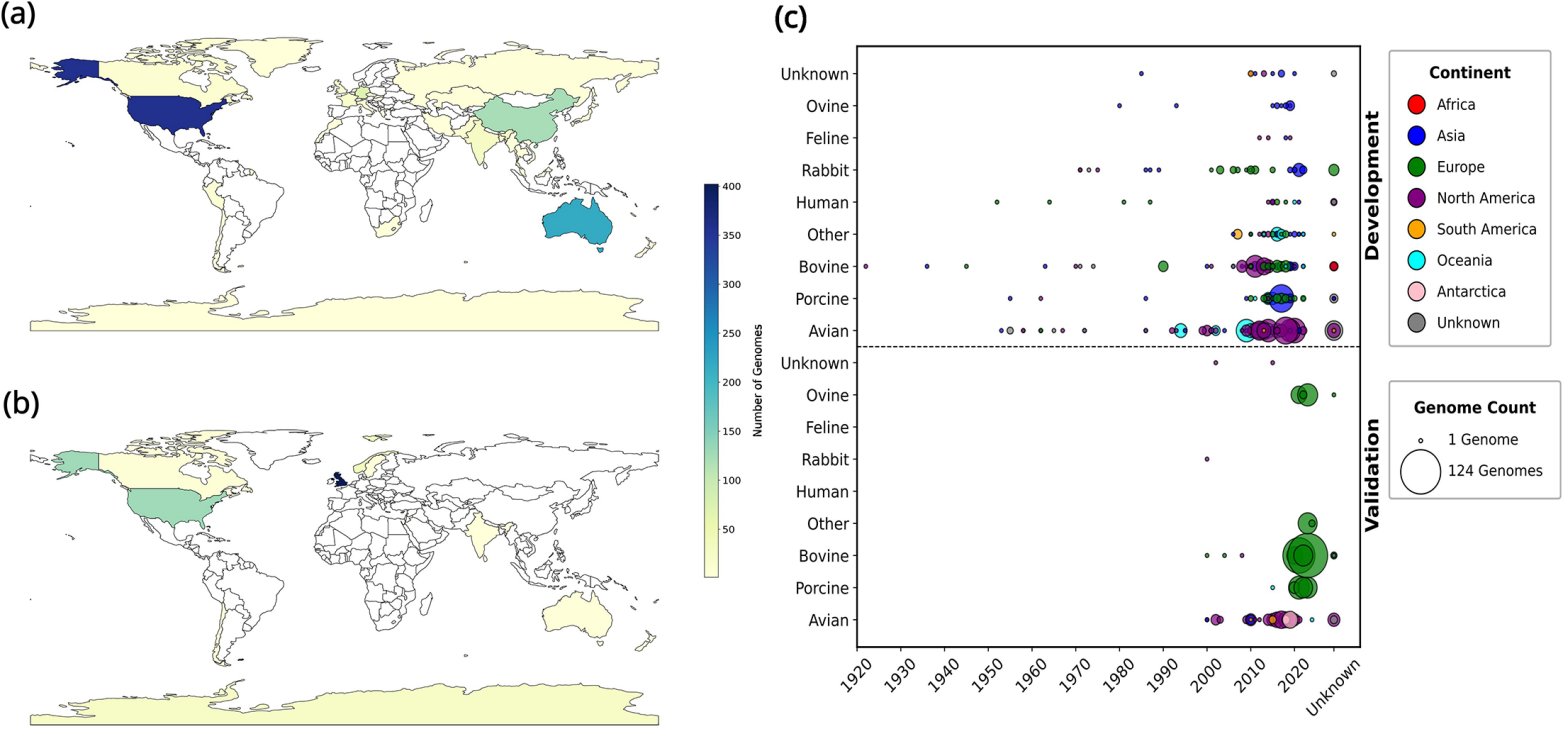
Temporal, host and geographic distribution of genomes in the development and validation datasets used for constructing the cgMLST scheme. (**a**) Geographic distribution of genomes in the development dataset and (b) geographic distribution of genomes in the validation dataset. Countries are shaded according to the number of genomes contributed, with darker blue tones indicating higher counts. (**c**) Bubble plot summarizing genome counts by host species (*y*-axis), year of isolation (x-axis; 1922–2024) and continent (bubble colour). The upper and lower sections represent the development and validation datasets, respectively. Bubble size is proportional to the number of genomes per host-continent–year combination.

*P. multocida* genomes in both datasets were globally distributed (Fig. 1a, b). In the development dataset, most genomes originated from North America (369/973), Oceania (222/973), Asia (190/973) and Europe (121/973) (Fig. 1a). Conversely, the validation dataset was dominated by genomes from Europe (440/620) and North America (134/620) (Table S1, Fig. 1b).

Both datasets encompassed a diverse range of host species, predominantly avian (development: 489/973; validation: 172/620), bovine (185/973 and 264/620, respectively) and porcine (141/973 and 98/620, respectively) (Fig. 1c). Collection dates ranged from 1922 to 2024, with peak collection years being 2017 (development: 101/973) and 2021 (validation: 135/620) (Table S1, Fig. 1c). The most frequent genome subset in the development dataset was from Asian porcine isolates collected in 2017 (*n*=48), whereas bovine isolates from Europe in 2023 (*n*=127) predominated in the validation genomes dataset (Fig. 1c).

Overall, the development dataset included greater temporal and geographic representation, while the genomes of the validation dataset consisted primarily of more recent isolates from European bovine, porcine and ovine, as well as North American avian sources (Fig. 1c).

### Defining a *P. multocida* cgMLST scheme

Core genome loci were defined as genes present in at least 99% of genomes in the development dataset. Most loci were present in 50–99% of the genomes in the development dataset, with a sharp drop approaching 100%. Loci at or above this threshold were present in at least 99% of genomes and were selected for inclusion in the cgMLST scheme (Fig. S4).

After manual inspection and curation, 159 loci were excluded (Table S4), resulting in a final cgMLST scheme of 1,233 genes. No paralogous genes were identified among the retained loci. Using this final scheme, a cgST was assigned to 957 of the 973 development genomes (98.35%) and 597 of 620 validation genomes (96.29%) (Table S1). Overall, 1,554 of 1,593 genomes (97.55%) received a cgST assignment, while 39 genomes could not be assigned due to high numbers of missing loci (36 with 26–99 missing loci and three with more than 100 missing loci) (Table S5). Among the 1,554 typed assemblies, a total of 1,304 distinct cgST profiles were identified (Table S5), compared to only 239 unique STs obtained using seven loci MLST methods (Table S1). In total, 114,324 unique core gene alleles were detected, with individual genes ranging from 4 to 331 alleles each (Table S6).

### Functional categorization and recombination analysis of the core genes

**Fig. 2.**
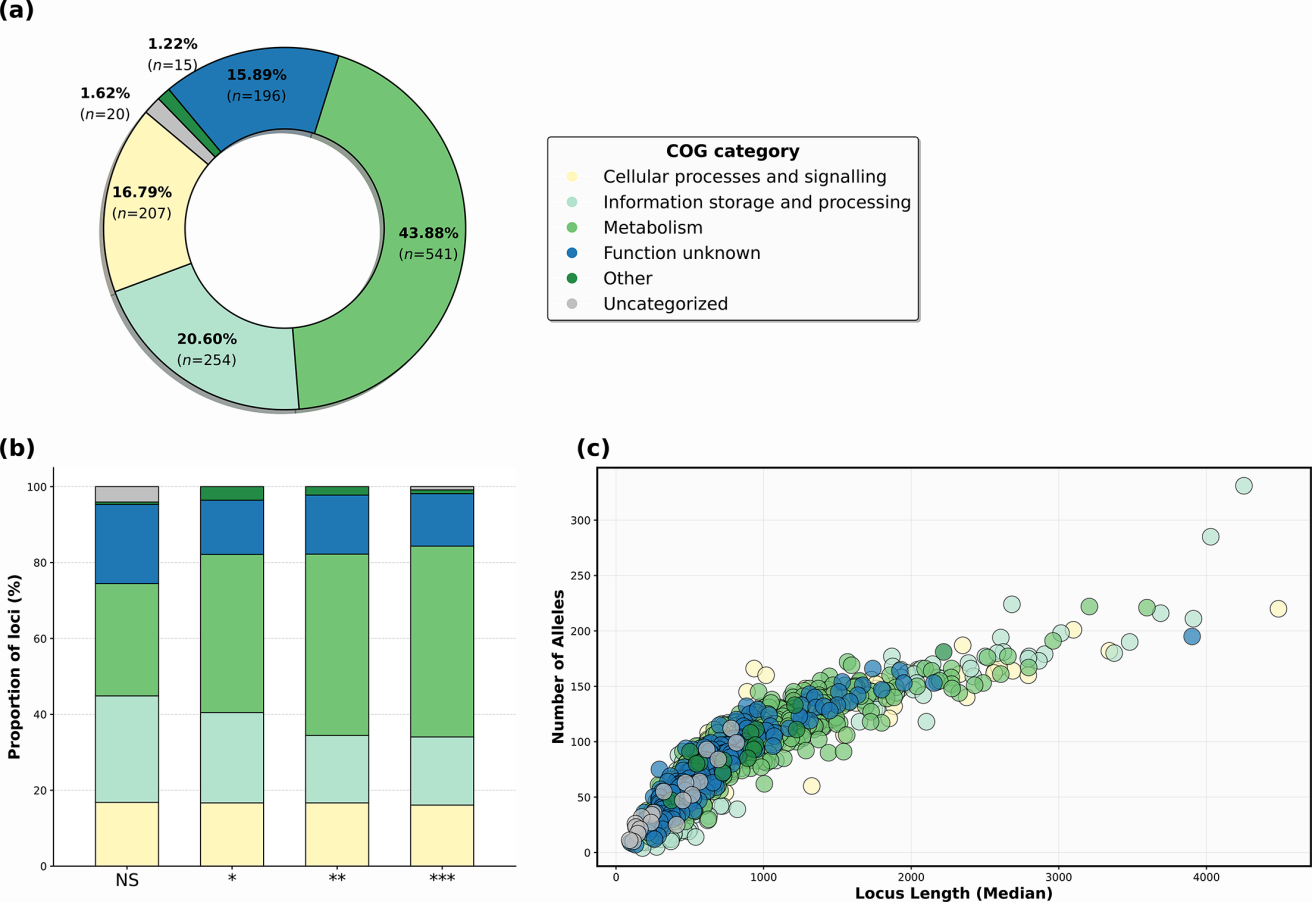
**(a**) Pie chart showing the functional classification of core genes using eggNOG-mapper and KEGG BRITE. Each gene was assigned a COG category and grouped into broader functional classes. (**b**) Genes were categorized according to their functional group and recombination significance levels: NS (non-significant), * (0.01<*P*≤0.05), ** (0.001<*P*≤0.01) and *** (*P*≤0.001). Bars represent the percentage of loci within each functional group across the four significance categories. (**c**) The number of alleles and the median length were calculated for each core gene. Genes were plotted and colour-coded according to their assigned functional group category. Donut chart with metabolism as the largest COG category at 43.88 percent. Stacked bars compare functional groups across recombination significance levels. Scatter plot shows a positive correlation between median locus length and allele count.

Functional annotation using eggNOG-mapper successfully assigned COG categories to 1,213 of the 1,233 core genes (98.37%). Among these annotated genes, 15 genes (1.22% of the total) spanned different functional groups, leading to their classification as ‘Other’ (Table S6, Fig. 2a)

The functional distribution of core genes revealed that the majority were involved in metabolism (43.88%), followed by information storage and processing (20.60%) and cellular processes and signalling (16.79%). Notably, 196 genes (15.89% of annotated genes) were classified as ‘function unknown’ (COG category S). To assess whether these genes of unknown function might represent overlooked AMR or virulence determinants, they were screened against the CARD and VFDB databases. This analysis revealed no AMR genes or virulence factors among these uncharacterized loci (Table S6, Fig. 2a).

Intragenic recombination events were assessed using the PHI statistic with *P*<0.05 considered statistically significant. Among the 1,233 core genes, 911 (73.88%) exhibited evidence of intragenic recombination, with 445 genes from the metabolism functional group showing significant recombination patterns (Fig. 2b). The high frequency of intragenic recombination in core genes increases allelic diversity, enhancing the resolution of the cgMLST scheme for strain typing and surveillance.

Core gene lengths varied considerably: 808 genes (65.53%) were <1,000 nt, 362 genes (29.37%) were 1,000–2,000 nt and 63 genes (5.11%) were ≥2,000 nt. Longer genes showed greater allelic diversity (Table S6, Fig. 2c).

### Defining the structure of *P. multocida* populations using the cgMLST scheme

To determine the optimal thresholds for the cgLIN code bins, we analysed the population structure of a subset of *P. multocida* (*n*=620) by evaluating pairwise allelic mismatches across cgMLST profiles. Each bin in the cgLIN code is defined by left (inclusive) and right (exclusive) thresholds based on the number of allelic differences when comparing cgMLST profiles. Pairwise comparisons were conducted across the subset (Fig. S5a), and to further resolve population structure, additional thresholds were set based on allelic mismatch distributions among isolates that belong to the same seven housekeeping gene STs, CCs, rST, capsular type and LPS serovar groups (Fig. S5b).

Clustering consistency and stability were assessed across thresholds, ranging from 1 to 1,233 allelic mismatches (0–100%). Optimal clustering was indicated by SI and W values approaching 1 (Table S7), confirming that clustering patterns in the 620-genome subset were consistent with the full population of 1,554 genomes. Both metrics plateaued between 197 (15.97%) and 910 (73.8%) allelic mismatches, as seen in Fig. S5a, b, indicating maximal stability and robustness in cluster assignment.

Two prominent peaks in allelic mismatches were observed in the pairwise comparisons shown in Fig. S5a, as well as in the pairwise comparisons of the taxonomic categories capsular type and LPS serogroup shown in Fig. S5b, guiding the selection of the first threshold, superlineage, at 903 mismatches, which defines the first bin of the cgLIN code scheme (Fig. S7).

For CC and ST groups, most pairwise comparisons involved fewer than 40% mismatches. A threshold of 419 mismatches (33.98%) was selected as the lineage threshold, as this value represented the optimal balance between consistency (SI) and stability (W). Isolates sharing the same rST exhibited high core genome similarity, with most having fewer than 196 mismatches (15.87%). This observation prompted the selection of a third threshold, designated as sublineage (Fig. S5b).

To further distinguish closely related *P. multocida* isolates, additional thresholds (T4–T12) were set at 6.24% (77 allelic mismatches), 4.70% (58 allelic mismatches), 2.83% (35 allelic mismatches), 1.29% (16 allelic mismatches), 0.56% (7 allelic mismatches), 0.32% (4 allelic mismatches), 0.16% (2 allelic mismatches) and 0.08% (1 allelic mismatch). Finally, a zero-mismatch threshold (T12) was established as each bin is bounded on the right by a threshold (exclusive) (Figs S5b and S7). The population structure derived from the 620 genomes dataset was compared against that obtained from 2 additional, randomly selected subsets of 421 and 901 genomes. Despite being independently selected, all three datasets displayed consistent patterns of pairwise allelic mismatches, with the same thresholds being identified across all subsets, further supporting the stability and robustness of the cgLIN code scheme (Fig. S6).

### Multilevel clustering of *P. multocida* genomes

**Fig. 3.**
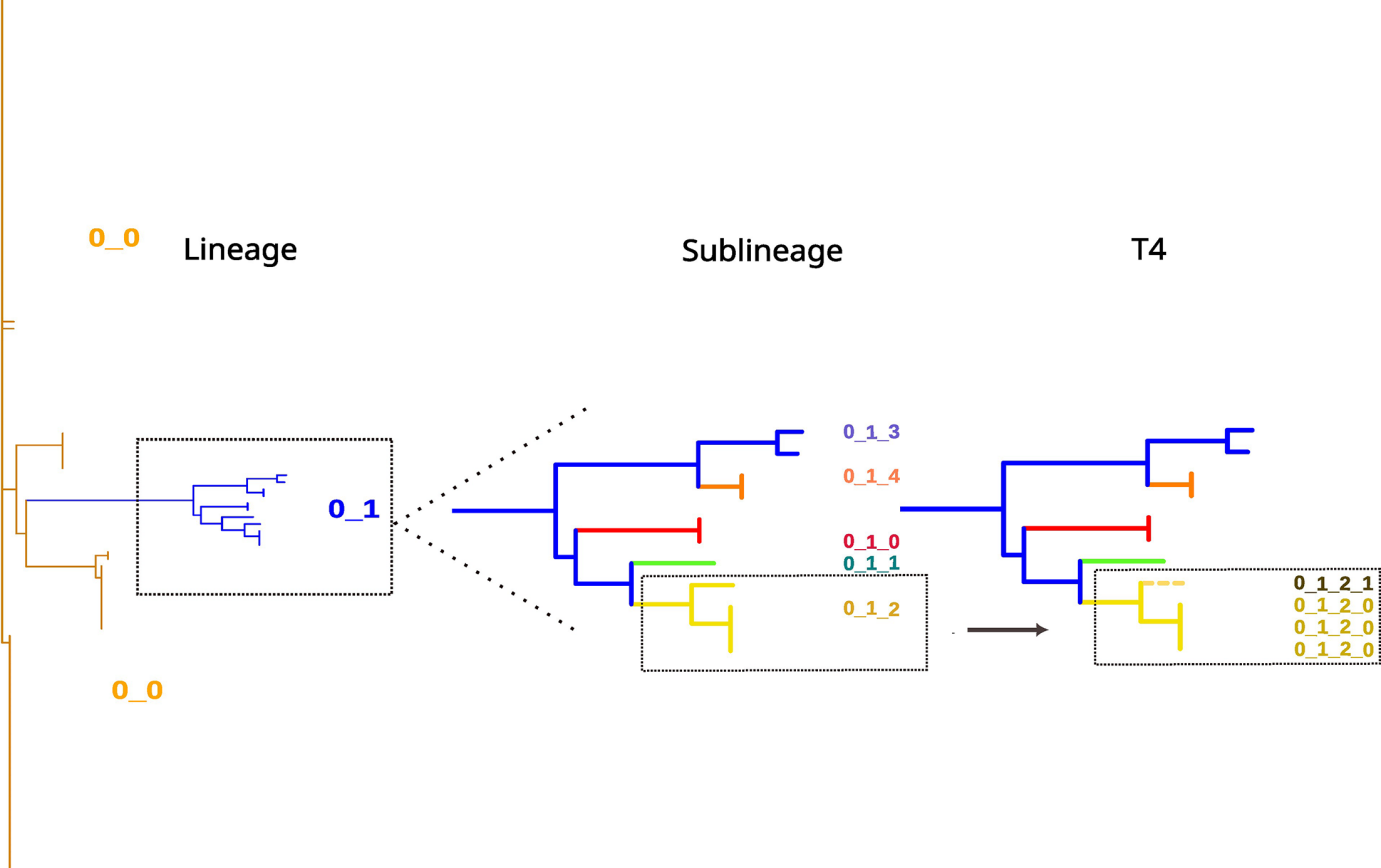
Illustration of phylogenetic relationships using superlineage cluster ‘0’ as an example. The figure shows the relationships among lineage clusters (left), sublineage clusters (middle) and T4 clusters (right), along with their corresponding LIN barcodes.

Among the 1,554 *P*. *multocida* genomes with assigned cgSTs, 1,304 unique cgST profiles were identified. To elucidate genetic relationships, cgLIN codes were assigned to all genomes, clustering them according to predefined allelic mismatch thresholds. The *P. multocida* LIN code was represented by a 12-digit numerical barcode, reflecting cluster assignments across hierarchical thresholds (superlineage, lineage, sublineage and T4–T12). The first position at which two cgLIN codes differ indicates the threshold at which the genomes are no longer clustered together. For example, a superlineage cluster ‘0’ comprises two lineage clusters: ‘0_0’ and ‘0_1’. The lineage cluster ‘0_1’ is further divided into five sublineage clusters: ‘0_1_0’, ‘0_1_1’, ‘0_1_2’, ‘0_1_3’ and ‘0_1_4’. The sublineage cluster ‘0_1_2’ is subdivided into two distinct T4 clusters: ‘0_1_2_0’ and ‘0_1_2_1’ (Fig. 3), resembling a tree-like hierarchical structure.

A total of 1,226 unique cgLIN codes were identified among the 1,593 *P*. *multocida* genomes (Table S8), resulting in 83 superlineage clusters, 115 lineage clusters, 169 sublineage clusters and 312 T4 clusters. The majority of superlineage, lineage and sublineage clusters included more than one genome: 58 of 83 (69.88%) superlineage clusters, 70 of 115 (60.87%) lineage clusters and 80 of 169 (47.34%) sublineage clusters. Notably, over half of all isolates were concentrated in a small number of clusters, with 4 superlineage clusters containing 810 genomes, 5 lineage clusters encompassing 841 genomes and 5 sublineage clusters including 816 genomes (Table S8).

To assess the concordance between cgLIN clustering and other established *P. multocida* classification methods, including ST, CC, rMLST, LPS type and capsular type, using the ARI, where a value of 1 indicates perfect agreement. At the lineage and sublineage levels, cgLIN clustering showed near-perfect concordance with CCs (ARI=0.99 for both levels). At the T4 level, cgLIN clusters were most consistent with STs, with an ARI of 0.83. Capsular and LPS types showed the least concordance across all cgLIN code levels (Fig. S8).

### cgLIN code validation

**Fig. 4.**
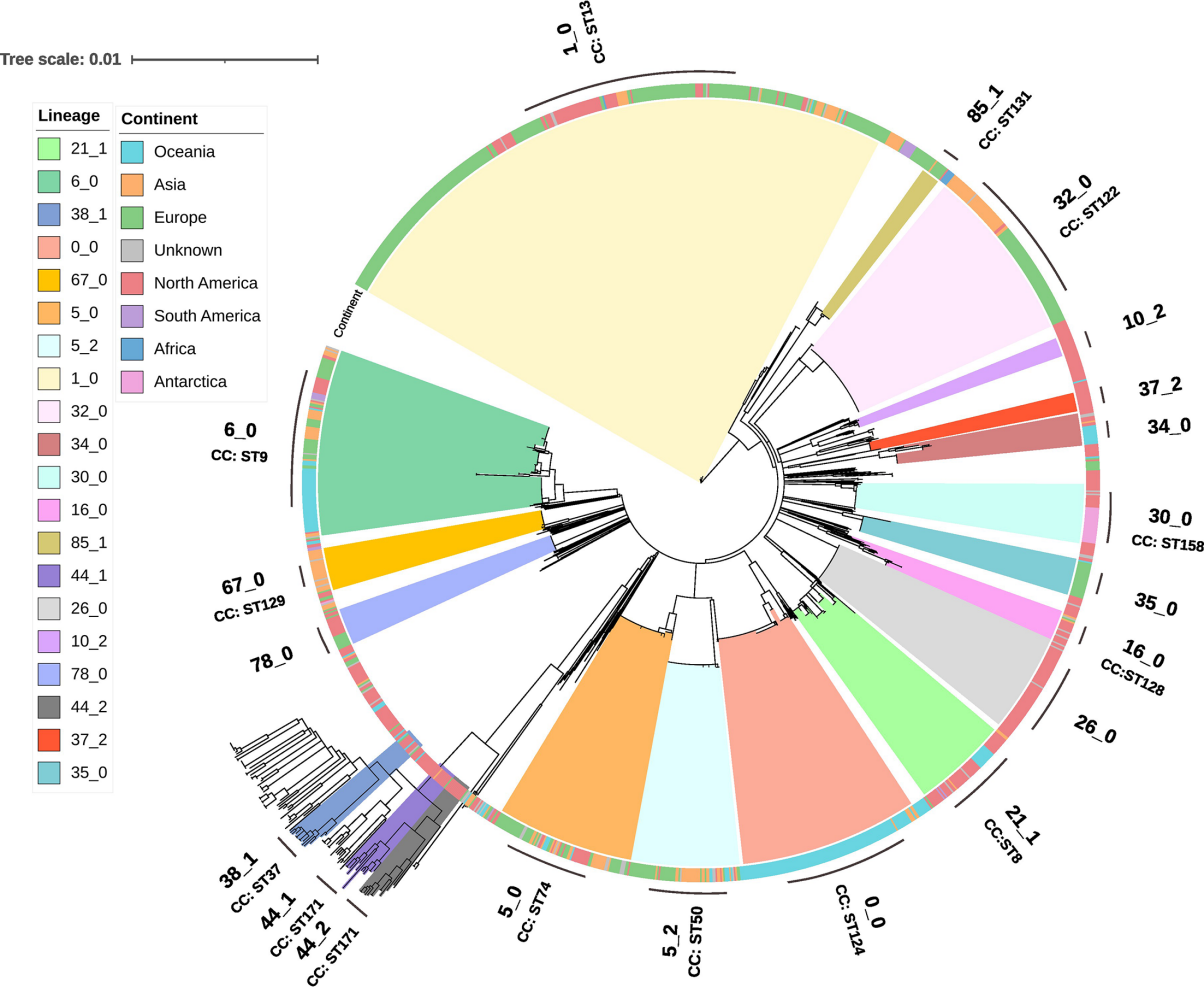
Phylogenetic tree of 1,554 *P*. *multocida* genomes based on a nucleotide alignment of 1,233 cgMLST loci. The 20 most prevalent lineage clusters are highlighted and annotated with their corresponding CC and the first 2 digits of the lineage clusters. The outer ring is colour-coded by the continent of origin of the isolates.

To further evaluate the congruence between cgLIN code-based clustering, core genome phylogeny and whole-genome k-mer tree, we reconstructed a phylogenetic tree based on nucleotide alignments of all 1,233 cgMLST loci (Fig. 4) and a k-mer-based tree for the 1,554 genomes with assigned cgMLST profiles (Fig. S9). The 20 most prevalent lineage clusters, collectively representing 1,272 of the 1,554 genomes (81.85%), were highlighted in the trees. These clusters formed well-defined, monophyletic groups. This close correspondence demonstrates that lineage-level clustering accurately captures major evolutionary relationships rather than producing arbitrary groupings (Figs 4 and S9). Further supporting this finding, we observed near-perfect concordance between lineage clusters and CC assignments (Fig. 4). Among the 115 lineage clusters, excluding those of unknown origin, 30 (26.09%) contained *P. multocida* genomes from multiple countries, whereas 85 (73.91%) included genomes from a single country (1–12 genomes). Moreover, 87 clusters (75.65%) comprised genomes from a single continent (1–66 genomes) (Table S8; Fig. 4).

### Example applications of the LIN code

**Fig. 5.**
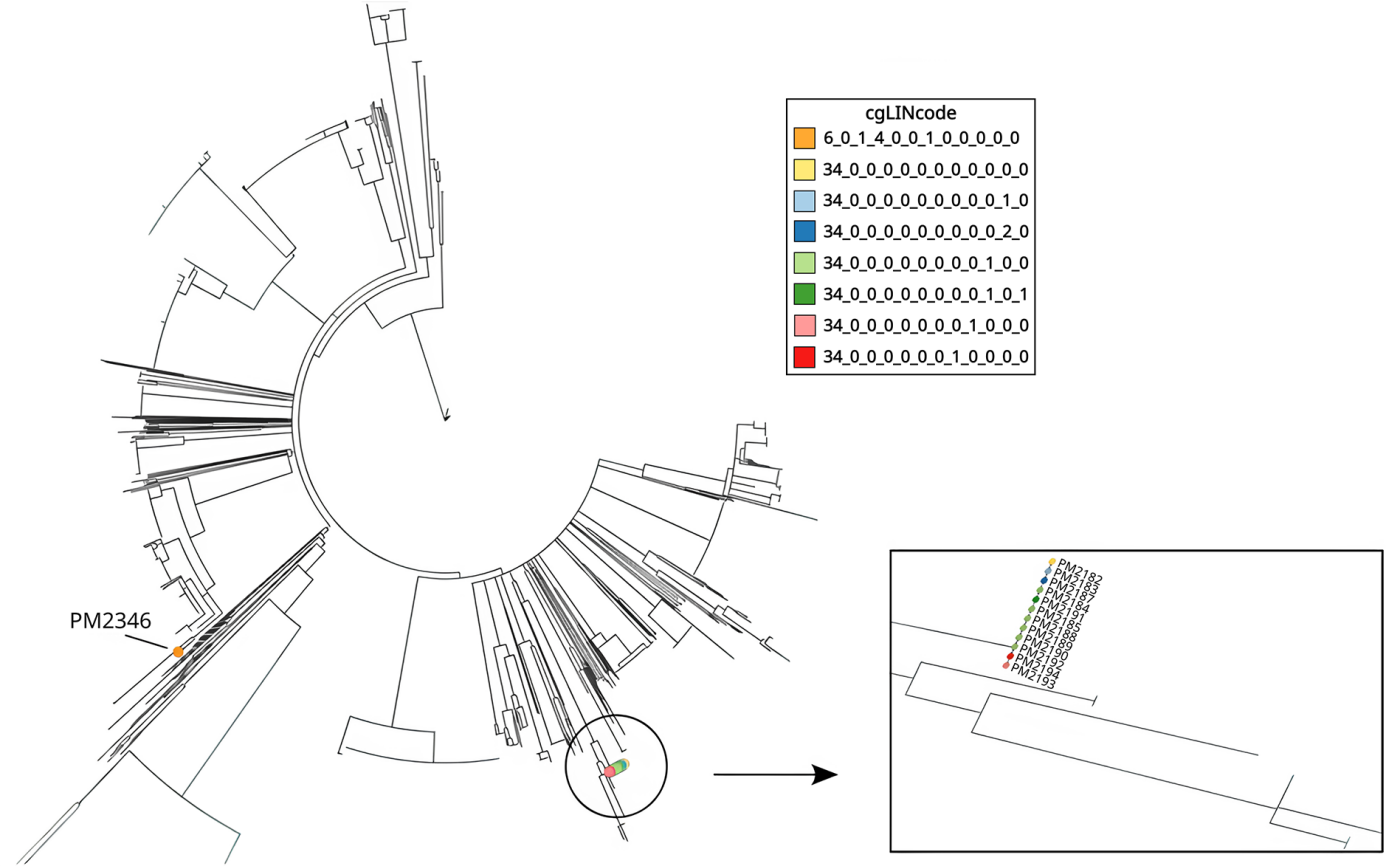
Phylogenetic tree of *P. multocida* isolates based on cgMLST loci, generated using the PubMLST phylogenetic tree tool and visualized with Microreact. Isolates are colour-coded according to their LIN code assignments. Isolate PM2346, previously reported in the original publication as unrelated to the outbreak, is indicated. Isolates originally associated with the outbreak are highlighted within a black circle.

To illustrate the cgLIN code scheme, we compared cgLIN code cluster assignments against previously published outbreak investigations of *P. multocida*. In the first case, isolates collected from an Australian zoo between November 2015 and February 2016, as well as in August 2017 (PM2346), were analysed [50] (Fig. 5). Allele sequences for the 1,233 cgMLST loci were aligned and visualized. The cgLIN code clearly distinguished the isolate PM2346, which was isolated in August 2017 from the outbreak cluster by assigning it to a different cluster at the superlineage bin (>903–1233 allelic differences) (Fig. S7), supporting its classification as unrelated to the outbreak, consistent with the original SNP-based analysis. All outbreak-associated isolates formed a single cgLIN cluster, sharing identical codes for the first seven bins and differing only in the eight, corresponding to allelic differences at ≤16 core loci. These findings matched the original reported core genome SNP analysis, which showed PM2346 as unrelated and outbreak isolates as highly similar (Fig. 5).

**Fig. 6.**
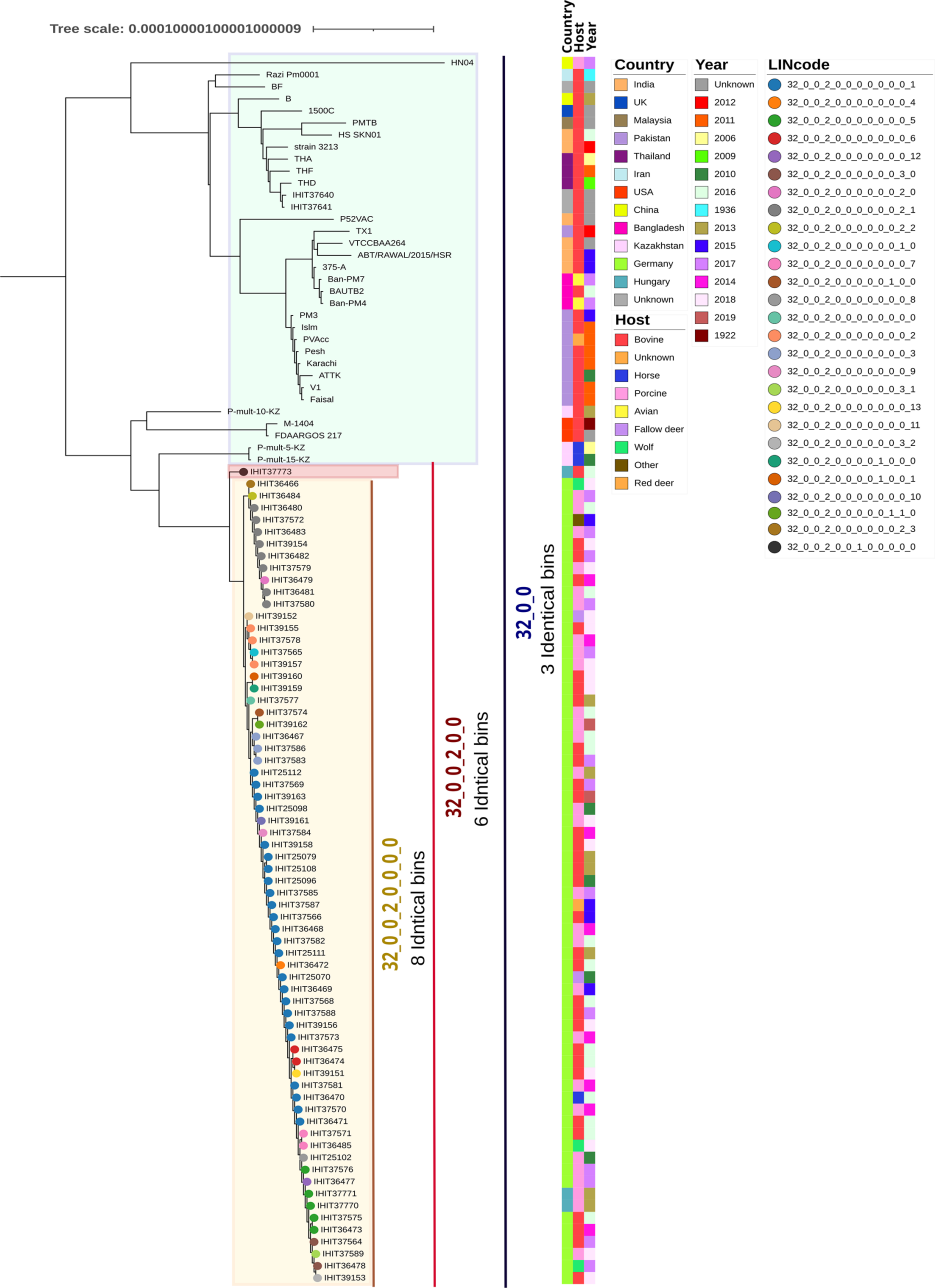
Maximum-likelihood phylogenetic tree constructed from core genome SNPs of 102 *P*. *multocida* isolates following a similar analysis pipeline to the original SNP-based analysis. Coloured circles represent distinct LIN codes assigned to each isolate. The clade includes 65 isolates from Germany and two isolates from Hungary (light yellow box) associated with an outbreak; these are distinct from one genetically separate Hungarian isolate (light red box). The globally distributed reference isolates form a separate clade (light blue). The blocks to the right of the tree indicate the country of origin, host species and year of isolation for each isolate.

A second example of the applicability of the cgLIN code was displayed using 102 *P*. *multocida* isolates for which SNP-based analysis had previously been conducted to assess phylogenetic relationships and identify outbreak isolates. For this dataset, we reconstructed a phylogenetic tree following a similar analysis pipeline to the original described in the original SNP-based study and coloured them according to the cgLIN code [51]. All isolates were associated with HS cases: 65 from Germany, 3 from Hungary and 34 additional global HS isolates [51]. According to the original study, all German isolates and two of the Hungarian isolates (highlighted by a light-yellow square in Fig. 6) clustered together, exhibiting only minor SNP differences among themselves but differing more substantially from a third Hungarian isolate, IHIT37773, which was located in the light red square. This genetic distinction was also reflected in cgLIN code clustering: the 65 German isolates and 2 Hungarian isolates shared identical cgLIN codes in the first eight bins and diverged only at the ninth bin (indicating allelic differences in ≤7 core genes) (Fig. S7). In contrast, the genetically distinct Hungarian isolate IHIT37773 diverged at the seventh bin compared to the 67 other isolates (reflecting allelic differences of >16–35 core genes) (Fig. S7), mirroring the SNP-based phylogeny results. Furthermore, the 68 German and Hungarian isolates were distinct from the 34 global HS reference isolates (highlighted in the light blue box in Fig. 6) by divergence at the fourth cgLIN bin (reflecting allelic differences of >77–196 core genes) (Fig. S7), consistent with the broader genomic differences observed in the SNP analysis of the original study [51].

Lastly, we demonstrated the capability of LIN code using 75 *P*. *multocida* ST20 isolates collected over 5 years during fowl cholera outbreaks on an Australian free-range broiler farm with published SNP-based analyses [52]. A phylogenetic tree was constructed under conditions similar to those of the original study, with different colours assigned to each cluster to reflect the distinct cgLIN codes assigned (Fig. S10). According to the original study, these isolates formed two major genomic clades (Fig. S10). Analysis of the cgLIN codes also separated the isolates into two major clades, with divergence observed at the sixth bin, corresponding to differences of >35–58 core gene alleles (Fig. S7). Within each clade, isolates were also clustered in different cgLIN clusters, as indicated by the assigned colours, with divergence observed at the 11^th^ and 12^th^ LIN code bins, when comparing them (Fig. S10).

## Discussion

Accurate and standardized bacterial classification is fundamental for infectious disease surveillance, particularly for pathogens of both human and animal health importance, such as *P. multocida*. The adoption of globally harmonized typing schemes enables consistent data interpretation across laboratories, facilitates the rapid identification of transmission pathways and supports the detection of outbreaks and epidemiological trends [9,25]. To strengthen *P. multocida* surveillance, we developed and validated a cgMLST scheme coupled with a hierarchical LIN code system.

The cgMLST scheme, based on 1,233 core genes, showed robust performance across 1,593 isolates from diverse hosts and geographic origins, successfully assigning cgSTs to 97.55% of genomes tested. Our estimate of the *P. multocida* core genome is consistent with previous pangenome studies, which reported between 561 and 1,806 core genes [59,63]. Differences across studies are likely attributable to dataset size, sequencing quality, assembly strategies [64,65] and the treatment of paralogous genes [66,68]. To minimize additional sources of bias, we also excluded low-quality assemblies and analysed a large, diverse dataset, yielding a stable estimate of the core genome. Beyond this robustness, this cgMLST scheme provides substantially enhanced discriminatory power compared to other typing approaches. It identified 1,304 distinct cgSTs, compared with only 239 STs defined by the 7-locus MLST scheme, demonstrating its superior resolution for global surveillance and the detection of novel variants.

When comparing the cgMLST-LIN code system with *P. multocida* capsular serogroup classification and LPS serotyping, it is evident that, although these methods are informative for cell surface antigens, they do not reliably reflect core genomic relationships. For example, the low ARI between serotype assignments and cgLIN code lineage clusters indicates that genomes sharing the same serotype are frequently distributed across distinct lineages. Conversely, individual lineage clusters often encompass multiple serotypes. Therefore, serotype prediction cannot currently be directly inferred from cgMLST or cgLIN code. However, the LIN code system showed near-perfect concordance with established MLST CCs at the lineage and sublineage levels (ARI=0.99). This high concordance is significant because MLST CCs are defined using slowly evolving housekeeping genes with minimal recombination, indicating that inclusion of recombinant core genes does not distort higher-level population structure. It also ensures continuity with historical surveillance datasets, facilitating integration with existing epidemiological data. Clusters assigned by the cgLIN code were further validated against SNP-based and k-mer phylogenetic analyses, showing agreement at the lineage level while offering a practical advantage. Unlike phylogenetic approaches that often require specialized bioinformatics expertise and substantial computational resources, cgLIN code integration with the BIGSdb platform and PubMLST infrastructure ensures global accessibility through standardized analysis pipelines [12].

Although 73.88% of core genes included in the scheme exhibited evidence of intragenic recombination events, this does not compromise the scheme. While intragenic recombination increases allelic diversity and enhances the cgMLST resolution, it can introduce homoplasy that may obscure true phylogenetic relationships in long-term evolutionary analyses. However, the gene-by-gene approach of cgMLST treats each allelic change as a single evolutionary event regardless of whether it arose by point mutation or recombination [9,14]. This approach reduces the impact of intragenic recombination compared to SNP-based methods, where multiple SNPs introduced by a single recombination event can inflate genetic distances.

In our practical applications, we observed that isolates from the same outbreak diverged from the eight LIN bin onwards, while isolates from different outbreaks differed at or before the fourth bin. Previous studies associated the fifth level onwards of the LIN code with clonal groups and subgroups, with the aim of differentiating very closely related pneumococci [19]. Nevertheless, outbreak delineation should not rely on fixed thresholds but should be interpreted in conjunction with epidemiological, temporal and spatial data. Beyond outbreak delineation the cgLIN code system offers additional practical advantages for surveillance networks. The use of numerical codes facilitates data sharing across institutions operating under different data protection regulations, as codes can be communicated without exposing raw genomic sequences, a feature particularly valuable for rapid international collaboration during emerging disease events.

Despite these advantages, we acknowledge several limitations that should guide implementation and interpretation. First, as with all sequence typing methods, profile assignment depends on assembly quality, and highly fragmented genomes may fail to receive a cgST or cgLIN code. A key technical consideration is that missing loci are excluded from pairwise allelic distance calculations, which can artefactually reduce the apparent distance between isolates. To mitigate this effect, we applied a threshold of 25 or fewer missing alleles (~2%) for cgST and LIN code assignment. This ensures that pairwise distances are calculated using the vast majority of core gene loci while minimizing the impact of assembly quality variations on LIN code stability. The 25-allele threshold represents the established PubMLST standard for recently developed cgMLST schemes and balances assignment robustness with maximizing successful cgST assignments while preserving population structure integrity [19,23, 24]. Second, like all MLST-based methods, cgMLST does not capture accessory genome variation, which may harbour traits relevant to virulence and/or AMR. Previous analyses demonstrated that AMR and virulence genes in *P. multocida* are commonly associated with mobile genetic elements rather than being vertically inherited through the core genome [2, 69,72]. For example, the *toxA* gene encoding dermonecrotic toxin resides within a prophage . These findings highlight the importance of integrating the cgLIN code system with AMR and virulence profiling in epidemiological investigations to provide comprehensive insights for vaccine selection and disease management strategies.

In conclusion, the cgMLST scheme implemented in the hierarchical LIN code system presented here provides a standardized, high-resolution framework for typing *P. multocida*. Combining the stability of cgMLST with the flexibility of multilevel LIN nomenclature enables efficient preliminary assessment of isolate relatedness, significantly enhancing outbreak detection, transmission tracking and strain typing for vaccine selection. We propose the adoption of this cgLIN system as a standard tool for *P. multocida* epidemiology, ensuring reliable strain classification and clear communication among researchers and public health authorities. Ultimately, this system strengthens global surveillance by providing standardized, comparable and accessible data through PubMLST.

## Supplementary material

10.1099/mgen.0.001733 Supplementary Material 1.

10.1099/mgen.0.001733 Supplementary Material 2.

## Abbreviations

AMR: antimicrobial resistance
APHA: Animal and Plant Health Agency
ARI: adjusted Rand index
CARD: Comprehensive Antibiotic Resistance Database
CC: clonal complex
cgLIN: cgMLST-based life Identification number
cgMLST: core genome multilocus sequence typing
cgST: core genome sequence type
COG: Cluster of Orthologous Genes
HS: haemorrhagic septicaemia
KEGG: Kyoto Encyclopaedia of Genes and Genomes
LIN: life identification number
LPS: lipopolysaccharide
MLST: multilocus sequence typing
PHI: pairwise homoplasy index
rMLST: ribosomal multilocus sequence typing
rST: ribosomal sequence type
SI: Silhouette index
ST: sequence type
VFDB: Virulence Factor Database
W: Wallace coefficient
WGS: whole genome sequencing
