# Supplementary material for: A standardized core genome multilocus sequence typing and life identification number barcoding framework for global Pasteurella multocida surveillance and outbreak investigation
Source: Microb Genom. 2026 Jun 25;12(6):001733. doi: 10.1099/mgen.0.001733 (PMC13308733; doi:10.1099/mgen.0.001733)
Supplement: Supplementary Material 1. [file mgen-12-01733-s001.pdf]

Supplementary Files

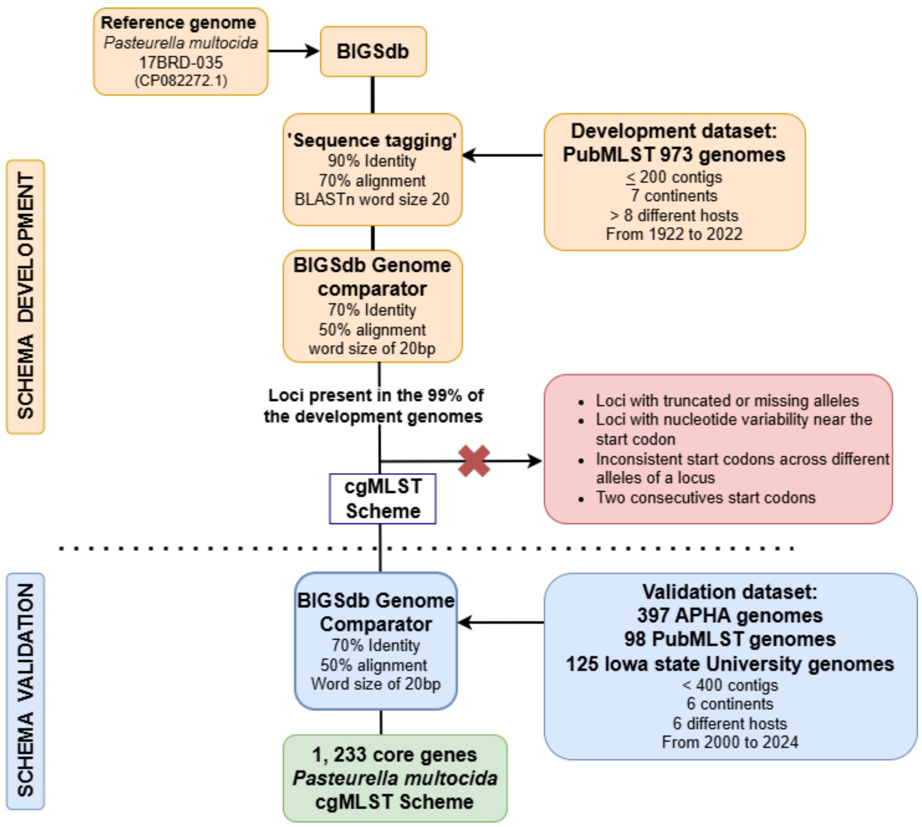

Supplementary Figure 1. Diagrammatic representation of the basic methodology used for: (i) Identification of core genes from PubMLST genomes, for the Development of the cgMLST scheme (top), and (ii) Validation of the scheme using APHA, PubMLST and Iowa State University genomes.

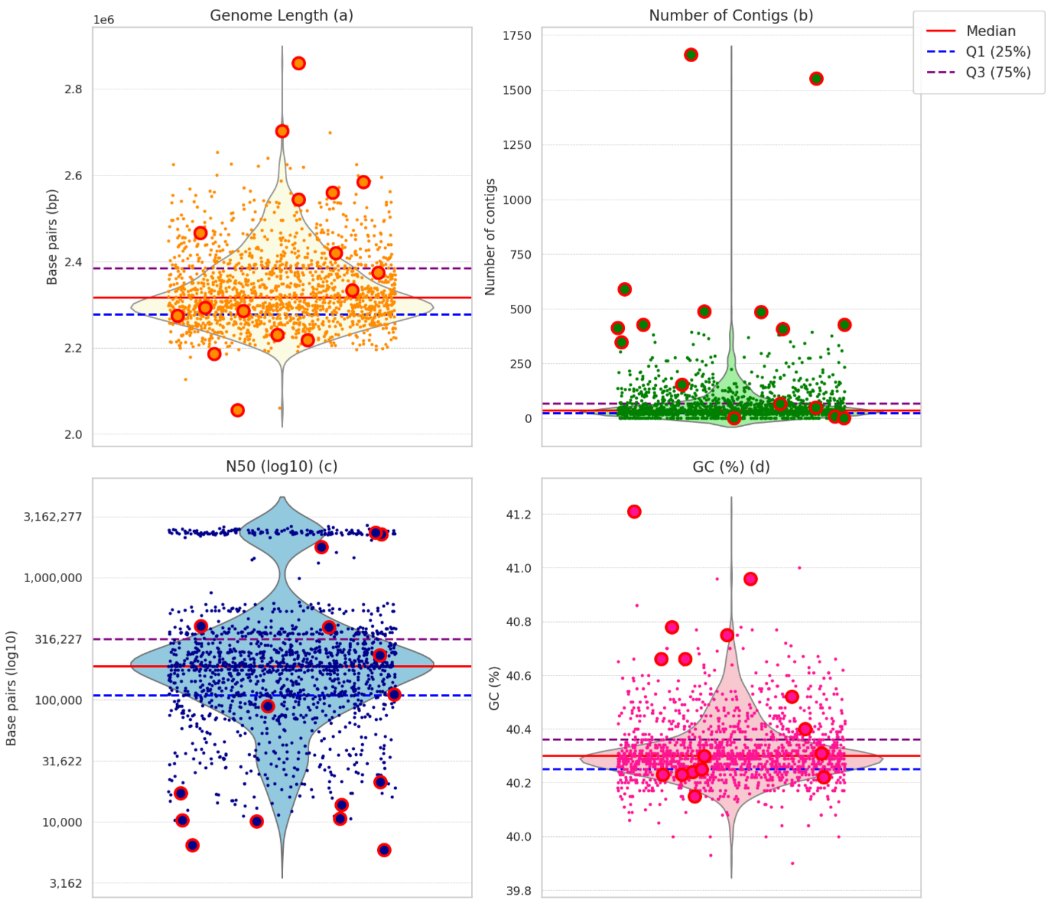

Supplementary Figure 2. Assembly quality metrics for all 1,593 *P. multocida* genomes included in this study. Violin plots presented the key assembly metrics for all the *P. multocida* genomes included in this study: a) genome length, b) number of contigs, c) N50, and d) GC (%) content. For each plot, the red line represented the median, while the blue and purple lines indicated the first and third quartiles, respectively. The width of the violin plot at any given y-axis value reflects the density of data at that value. Large, red-outlined dots correspond to assemblies excluded from the final dataset. Violin plots were generated using Seaborn.

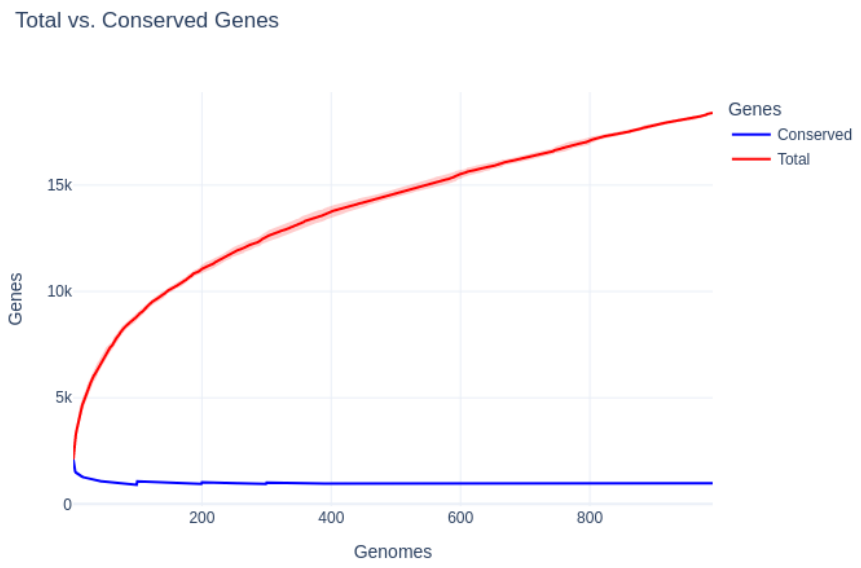

Supplementary Figure 3. Trend in Conserved and Total genes count with increasing number of *P. multocida* genomes analysed using Roary. The x-axis indicates the number of genomes (0 – 973), and the y-axis shows the number of conserved (core, blue line) and total (pangenome, red line) genes. Shaded areas represent the standard deviation from 10 random permutations of genome order.

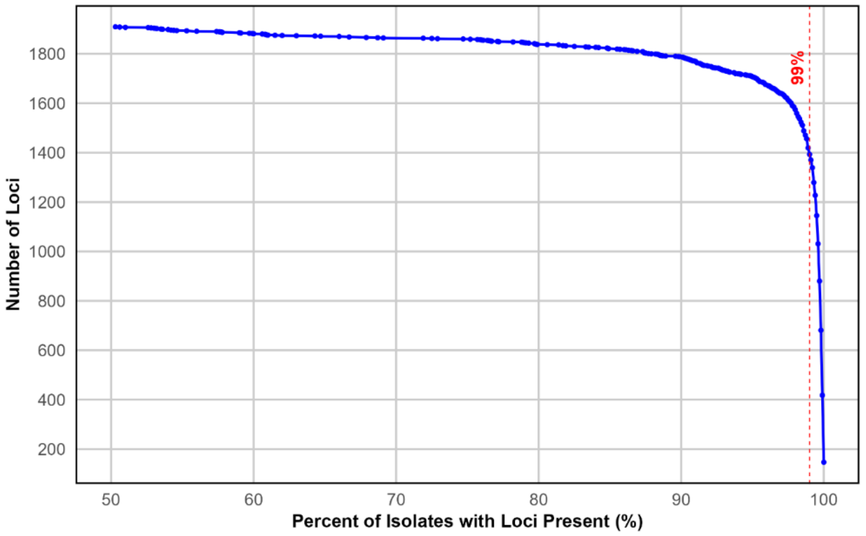

Supplementary Figure 4. Distribution of loci presence in the Development dataset. The x-axis shows the percentage of genomes in which each locus is present; the y-axis shows the number of loci. The red dashed line at 99% indicates the core gene threshold used in this study.

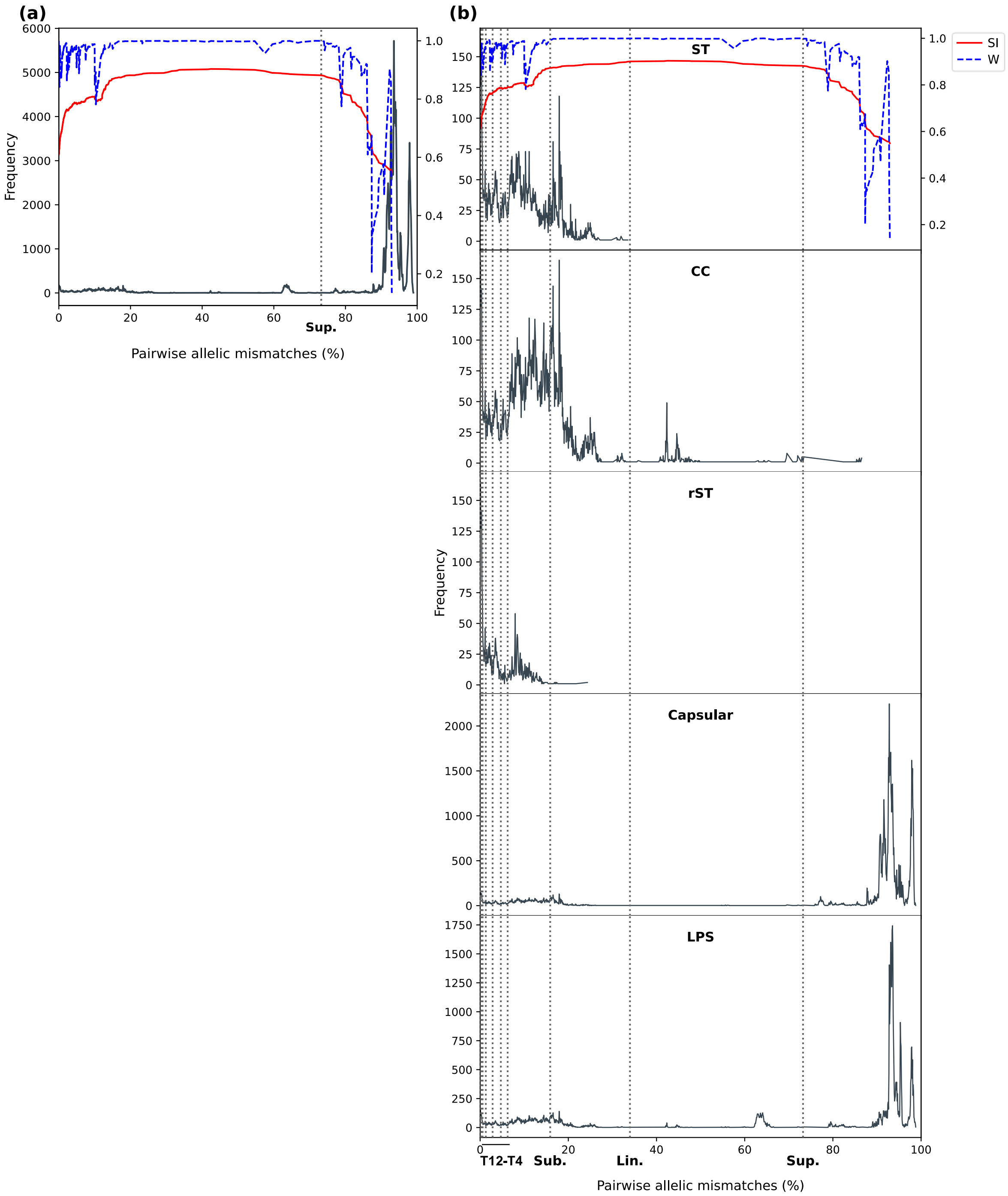

Supplementary Figure 5. Distribution of pairwise cgMLST allelic mismatches in *P. multocida*. a) Frequency distribution of pairwise cgMLST allelic differences among 620 *P. multocida* genomes. The x-axis represents the percentage of allelic mismatches relative to 1,233 core genome loci. The Silhouette Index (SI; red solid line) and Wallace Coefficient (W; blue dashed line) are overlaid on the right y-axis, with values approaching 1 indicating optimal clustering stability. b) Distribution of pairwise allelic differences for the 620 genome pairs within the same taxonomic groups (rST, ST, CC, capsular type, and LPS serovar). Vertical dotted lines indicate the defined cgLIN code

thresholds from finest to broadest resolution: T4–T12, Sublineage (Sub.), Lineage (Lin.), and Superlineage (Sup.).

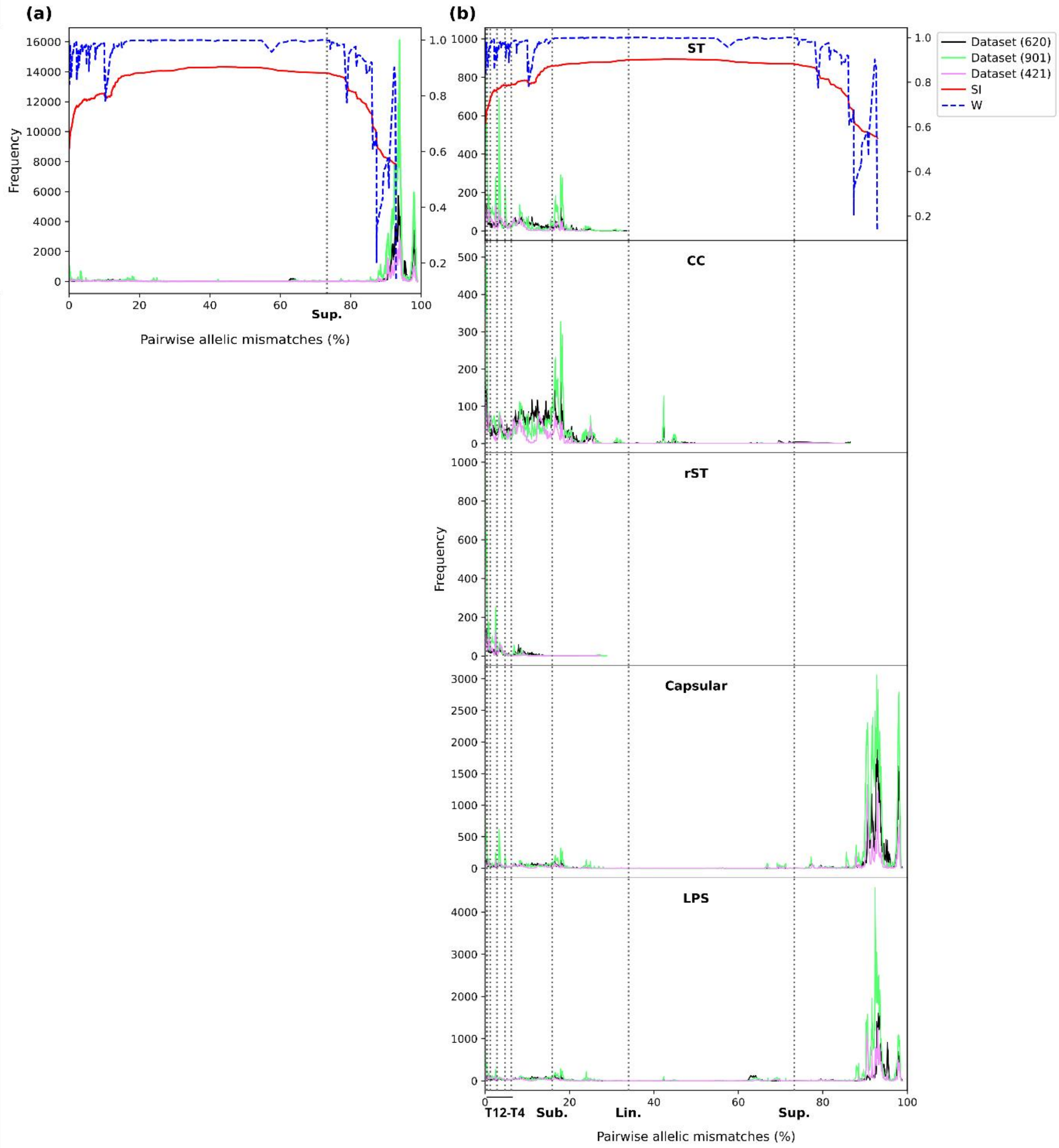

Supplementary Figure 6. Distribution of pairwise cgMLST allelic mismatches in *P. multocida*. a) Frequency distribution of pairwise cgMLST allelic differences among three datasets: 421 genomes (randomly chosen), 620 genomes (ensuring balanced representation of CCs), and 901 genomes (randomly chosen). The x-axis represents the percentage of allelic mismatches relative to 1,233 core genome loci. The Silhouette Index (SI; red solid line) and Wallace Coefficient (W; blue dashed line) are overlaid on the right y-axis, with values approaching 1 indicating optimal clustering stability. b)

Distribution of pairwise allelic differences for the 421, 620, and 901 genome pairs within the same taxonomic groups (rST, ST, CC, capsular type, and LPS serovar). Vertical dotted lines indicate the defined cgLIN code thresholds from finest to broadest resolution: T4–T12, Sublineage (Sub.), Lineage (Lin.), and Superlineage (Sup.).

Threshold	Sup.	Lin.	Sub.	T4	T5	T6	T7	T8	T9	T10	T11	T12
Allelic mismatches (Bins thresholds):	903	419	196	77	58	35	16	7	4	2	1	0
Bin number:	1	2	3	4	5	6	7	8	9	10	11	12
Genome A	0	0	0	0	0	0	0	0	0	0	0	0
Genome B	0	0	0	0	0	0	0	0	1	0	0	0
Genome C	0	0	0	0	0	0	0	0	1	0	0	0
Genome D	0	0	0	0	0	0	0	0	1	0	1	0

Supplementary Figure 7. Table of cgLIN code bin numbers, delimited by selected thresholds (Superlineage (Sup.), Lineage (Lin.), Sublineage (Sub.), T4 – T12). Each bin is defined by left-inclusive, right-exclusive mismatch intervals. The cgLIN code scheme begins by assigning a full-0 LIN code to the first cgST (Genome A), chosen randomly as the reference. Each subsequent genome is assigned a LIN code based on its allelic distance to the closest previously encoded genome, according to defined bin thresholds. For example, Genome B, which differs from the reference Genome A at more than 4 and up to 7 loci in the cgMLST profile, is assigned a 1 in Bin 9. Genomes with identical cgMLST profiles receive the same LIN code, while genomes with distinct profiles are assigned new codes at the appropriate bin level.

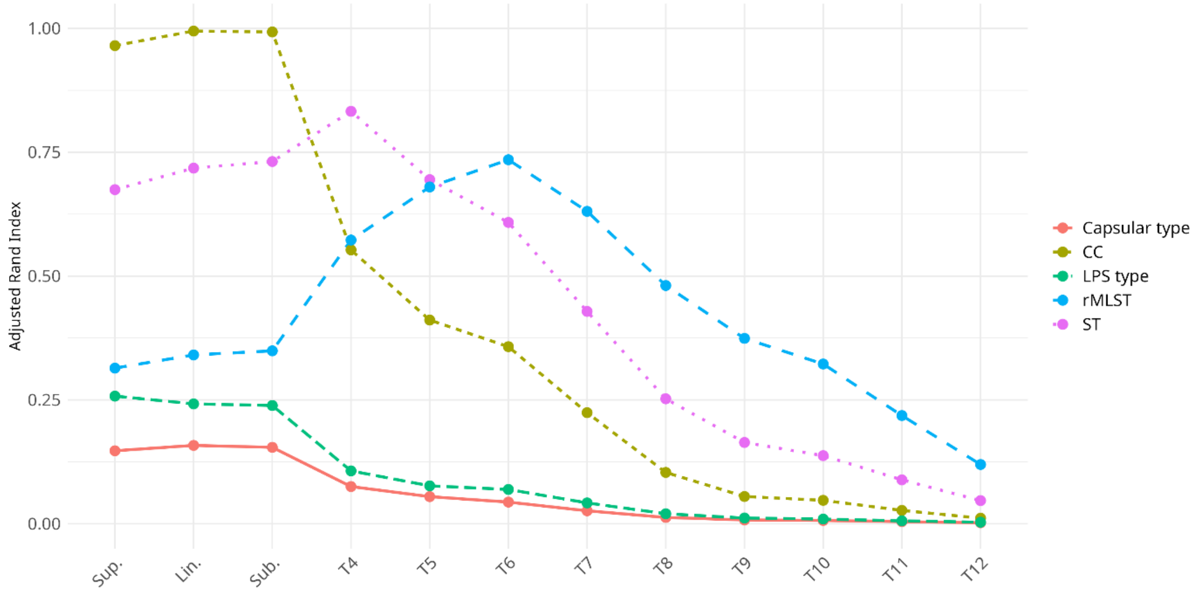

Supplementary Figure 8. Concordance between cgMLST LINcode cluster classification

levels (Superlineage (Sup.), Lineage (Lin.), Sublineage (Sub.), and T4 - T12) and other population classification schemes for *P. multocida*, measured by the ARI. A value of 1 indicates perfect concordance.

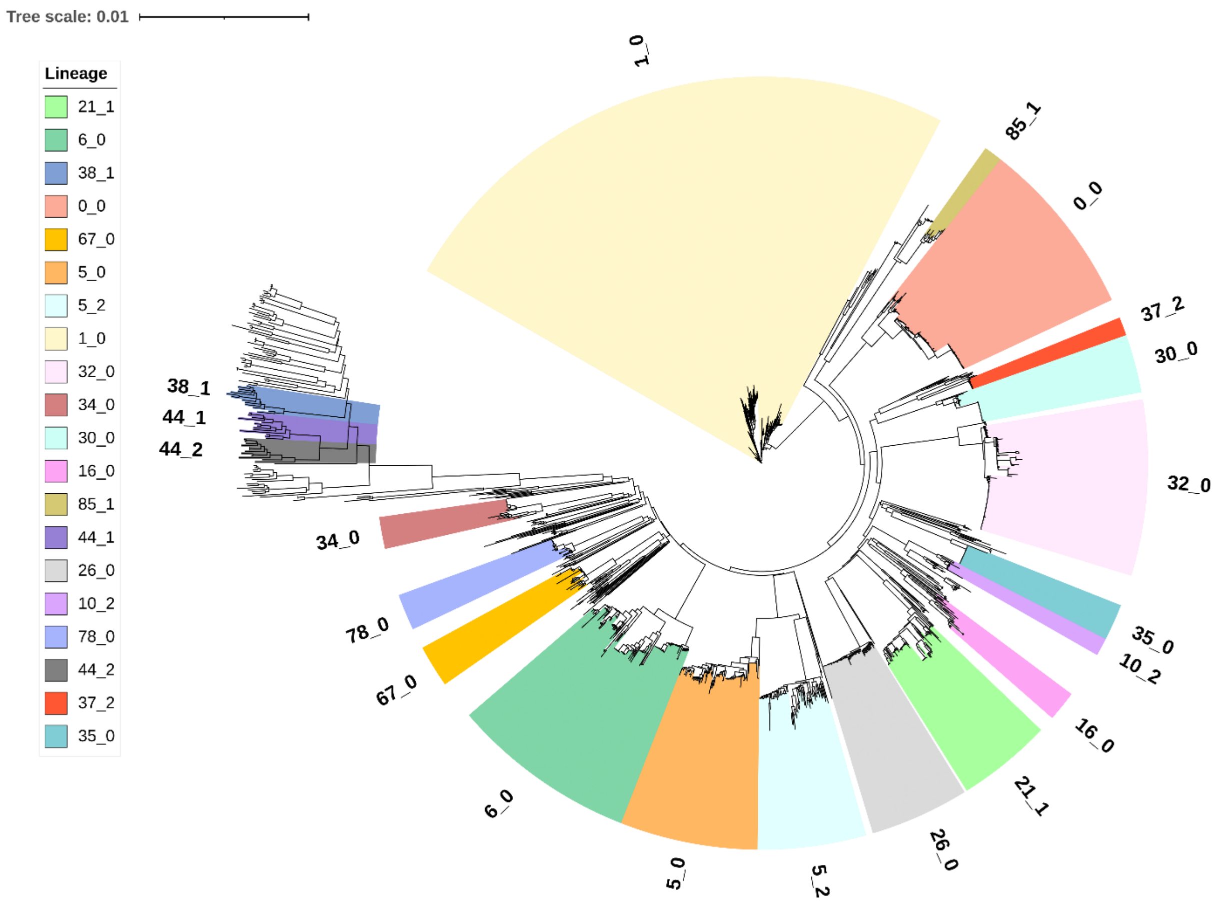

Supplementary Figure 9. Mash tree based on the whole-genome k-mer of 1,554 *P. multocida* genomes. The 20 most prevalent Lineage clusters are annotated with the first two digits of the Lineage LIN barcode.

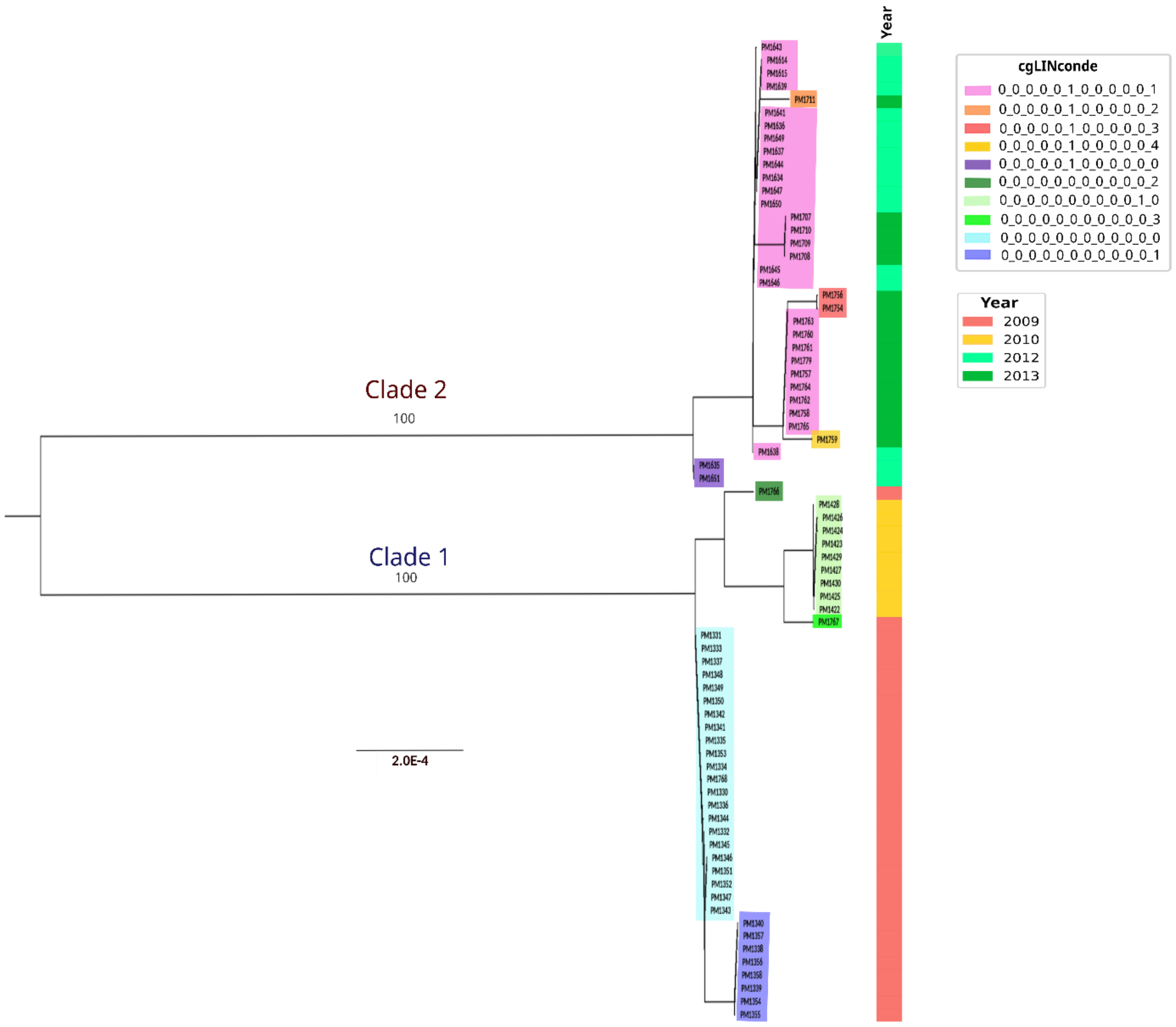

Supplementary Figure 10. Maximum likelihood phylogenetic tree based on core genome SNPs of 75 *P. multocida* isolates collected from an Australian broiler farm over five years. The tree was midpoint rooted. Two main clades (Clade 1 and Clade 2) were supported by 100% bootstrap values. Isolates were coloured within the tree based on their cgLIN code. The coloured bars on the right indicate the year of isolation.
